# Emotional states affect steady state walking performance

**DOI:** 10.1371/journal.pone.0284308

**Published:** 2023-09-14

**Authors:** Abhishesh Homagain, Kaylena A. Ehgoetz Martens

**Affiliations:** Faculty of Health, Department of Kinesiology and Health Sciences, University of Waterloo, Waterloo, Ontario, Canada; Kennedy Krieger Institute/Johns Hopkins University School of Medicine, UNITED STATES

## Abstract

Gait is a large component and indicator of health. Many factors affect gait including age, disease, and even mood disorders. Few studies have looked at the influence of emotional states on gait. This study aimed to investigate the influence of emotional states on walking performance to understand whether an emotional state may be an important factor to consider when evaluating gait. Thirty-six young adults were recruited (23F, 13M) and performed a neutral baseline condition of walking which included six passes of walking across an 8m walkway (a total of 48m of walking). Participants then completed 6 pseudo-randomized emotional state induction conditions while immersive 360-degree videos were used to induce the following emotional conditions: happiness, excitement, sadness, fear, and anger. Participants viewed the emotion elicitation videos using a virtual reality head-mounted display (HMD), then rated their emotional state using self-assessment manikins and walked (without the HMD) over a pressure sensor walkway. One-way repeated measures ANOVA and pairwise comparisons were used to examine differences in gait parameters across the emotional conditions. Participants walked with significantly reduced step length and speed during the sadness condition compared to the other emotional conditions and the neutral condition. Furthermore, participants adjusted the timing of their walking during the sadness condition and walked with significantly increased step, stance, and swing times compared to other emotional conditions, but not the neutral condition. Step time was significantly reduced during the conditions of excitement and fear compared to the neutral condition. Emotions may impact variety of gait parameters involving pace and rhythm, however have little influence on gait variability and postural control. These results indicate that perhaps the emotions of sadness and excitement should be taken into account as potential confounds for future gait analysis.

## Introduction

Human gait is a complex behaviour that is both dynamic and adaptable. It is achieved through bidirectional interactions between the motor system of the brain and other cortical and sub-cortical structures related to cognitive-emotional functions [1,2]. A growing body of research has focused on understanding the influence of emotion on gait. For example, several studies have found that depressed individuals demonstrate marked changes to spatiotemporal gait parameters including reduced velocity, stride length, and increased double limb support, and gait variability compared to controls [3–6]. Likewise, similar reductions to gait speed and step length as well as increases in step length variability and step time variability were also noted in highly anxious Parkinson’s patients compared to low anxiety Parkinson’s patients and age matched controls [7]. Whilst evidence suggests that mood disorders may give rise to discriminative changes in gait, less work has examined whether similar changes to gait can be induced by alterations in emotional state.

Previous work has investigated the influence of emotion on gait initiation (GI), which is the transient phase between a static position and locomotion [8]. GI encompasses the initial phase of gait, where anticipatory postural movements are performed in order to propel the body’s mass forward towards steady-state gait (which is achieved over a period of 3–5 steps) [8,9]. Studies have shown that emotional states can influence this initial phase of gait. Studies have shown evidence of increased velocity of the first step of walking when looking at pleasant pictures with high and low arousal compared to unpleasant pictures with low arousal [10]. Pleasant stimuli also showed increase in velocity of the center of pressure (COP) movement during the anticipatory postural adjustment phase compared to unpleasant stimuli which implicates emotional states and its modulation of motor action in whole-body movements [10–12]. Stins et al. [13] show that gait was initiated faster when initially seeing pleasant images and participants showed a reduction in step length when looking at unpleasant images compared to pleasant images. Using memory recall to elicit emotional states, Fawver et al. [14] showed evidence that happy and angry memories resulted in increased anterior velocity of the COP compared with sad memories.

Beyond (GI), steady state gait also has shown to be influenced by emotional states. Steady state gait is important to differentiate from GI as this state of walking is often used to report the general gait ability of an individual [15]. Previous work has shown that emotional states such as happiness and anger feature faster walking with an increased stride length, and increased step count—whereas other emotional states such as sadness and fear feature a reduction in speed, stride length, and step count [16–18]. These studies all used some form of the recall method to elicit emotions (where participants recall a time when they felt certain emotions). They also used a sample of exclusively professional actors [17], mix of professional actors and naïve subjects [18], and a mix of naïve subjects and non-professional actors [16]. Using music to elicit emotional changes, Park et al. [19] showed that pleasant music resulted in increased velocity, cadence, stride time and length compared to unpleasant music. Much of this past work has focused on quantifying aspects of pace but has done little work in measuring variability of gait even though recent research has emphasized the clinical relevance of gait variability [20,21]. Gait variability is an important determinant in evaluating risk factors such as fall risk and can even be a more sensitive predictor of falls than gait speed [22] and can even help with the detection of precursors to neurological diseases such as Parkinson’s Disease (PD) [23].

Furthermore, it is also important to note that previous research in this area [16–18] has used a sample of professional actors to quantify emotional differences in walking. This has been argued as a source of bias which could exaggerate gait results given that skilled actors are highly trained to produce stereotyped expressions [16]. Roether et al. [16] argued against using professional actors due to this reason and thus used non-professional actors instead in their study. Barliya et al. [18] also show that there is a difference in the smoothness of movements, thigh elevation angle and foot elevation angle between the professional-actor and non-actor groups. Although it was not reported as a significant difference, there was a difference in gait speed between the professional actor and non-actor groups reported by in the emotional states of anger and fear [18], however this was not further discussed by the authors. There were also differences in variability of gait speed across the two groups but again, this was not explained in detail by the authors. Finally, Halovic & Kroos [17] also highlight that some actors were better than others at portraying specific emotions and these differences were not uniform across emotions, leading again to the question of validity in the similarity of an actor’s movements compared to a non-actor subject. Thus, there remains a gap in understanding the influence of emotional states on steady state gait behaviour in non-actor populations across a wider range of gait parameters. It is imperative to understand the changes in gait characteristics that arise from fluctuations in emotional states; as these changes can possibly confound the gait characteristics observed in both healthy and non-healthy populations, leading to potential false characterizations of gait.

The main objective of this study is to measure the effect of emotional states on steady state gait characteristics in healthy young adults with no acting experience using a broader set of gait parameters. It was hypothesized that emotions such as happiness, excitement and anger would result in increased gait speed and step length compared to the neutral control emotion [16–18]. Consequently, emotions such as fear and sadness were expected to result in the opposite, showing decreased gait speed [1,16]. Finally, based on past work in clinical populations anxiety has shown to increase gait variability in PD patients [24]. Although fear and anxiety are separate conditions, they share similarities in the sense that fear is a key component of anxiety disorders and both are experienced in a response to a near or future threat [25,26]. Thus, it was hypothesized that fear would also lead to an increase in gait variability.

## Methods

### Participants

Thirty-six (n = 36) healthy young adult participants from the University of Waterloo were recruited for this study, however only a subset of twenty-three (n = 23) were used for further analysis (Table 1). Exclusion criteria included any previous difficulty experienced with virtual reality (VR) such as nausea, lightheadedness, fatigue etc. Recent history (6 months prior) of physical injuries that impacted gait, use of assistive devices for walking, or clinical diagnosis of mood disorders were also part of the exclusion criteria. Any participants taking medication that may induce or attenuate emotions were also excluded from the study. G*Power3 (Version33.1, Universitat Dusseldorf, Dusseldorf, Germany) was used to determine sample size [17]. Effect size from gait speed was used as the key dependent variable as it is a reoccurring parameter measured across many of the studies. As previous studies investigating emotion and gait have reported large effect size in gait speed, (η^2^ = 0.64 & η^2^ = 0.395 by Halovic & Kroos [17] and Park et al. [19] respectively) our sample size estimation used the threshold η^2^ = 0.14 for a large effect size to calculate the required sample size. Using an alpha error probability of 0.05, power of 0.8, and the effect size of 0.14, a sample size of 18 was calculated in G*Power3 [17]. The study was approved by the University of Waterloo Research Ethics Board. All participants provided written informed consent to participate in the study prior to collection. A virtual reality simulator sickness questionnaire (VRSQ) was used pre and post collection to account for possible motion sickness due to the VR environment.

**Table 1:** Demographic information.

Demographic Information	N = 23
Sex, N (%)	16F (69.5%) | 7M (30.5%)
Age (Mean, Range)	24(20–32) yrs
Height (Mean, Range)	166(9.32)) cm
mDES -Positive Emotions (Mean, SD)	2.77 (0.88)
mDES-Negative Emotions (Mean, SD)	1.21 (0.97)
Pre VRSQ-Score (Mean, SD)	3.73 (5.45)
Post VRSQ-Score (Mean, SD)	5.10 (6.36)

Demographic information of the n = 36 sample of recruited participants.

### Experimental procedure

After initial demographic, eligibility and consent information was collected, a modified differential emotional questionnaire (mDES) was used to screen initial emotional states of participants. Participants were then outfitted with an HTC Vive head mounted display (HTC, USA). VR content was generated using Unity (Unity Technologies, SF, CA, USA) and immersive videos were embedded inside the VR environment to induce an array of emotional states using Unity.

Work by Marin-Morales et al. [27] and Higuera-Trujillo et al. [28] have shown that VR environments can elicit similar physiological and psychological responses to real-life scenarios and support the use of VR in emotional elicitation paradigms; as this medium can take advantage of increased presence and immersion compared to traditional emotion elicitation methods such as pictures [27,28]. Similar studies as conducted by Li et al. [29] have used 360° videos (immersive videos) for emotion elicitation whilst recording self-reported measures of pleasure and arousal. Their results showed that the use of 360° video had similar pleasure and arousal scores to traditional emotion elicitation methods such as the International Affective Picture System (IAPS). This study used videos from the repository of videos created by Li et al. [29] as it had been previously used in VR scenarios, specifically for emotional elicitation [30].

Participants were given time to explore and navigate the virtual environment to familiarize themselves with the novel stimuli prior to collection. After familiarization was completed, participants performed three neutral walking trials. Walking trials involved 6 passes of walking across the 6m gait carpet resulting in approximately 40–60 total steps captured. To account for gait acceleration, participants started their walks 1m before the carpet and walked an additional 1m past the edge of the carpet on each pass, resulting in a total walk distance of 8m per pass (48m total). These number of passes was chosen to ensure that enough steps would be recorded to get valid measurements gait parameters [31]. All walking trials occurred in the real-world while the VR was used only for emotion elicitation. The emotional blocks were always completed after the neutral walking trials. Video of the participants on each walking trial was recorded during collection but was only used to confirm results from the pressure sensor carpet and was not used in any subsequent analysis.

Five emotional states of happiness, excitement, sadness, fear, and anger were elicited in a pseudorandomized blocked design. The blocks started with watching a video, then completing a pre-walk self-assessment manikin (SAM–which evaluated arousal, valence and dominance), followed by the walking trial, and culminating in the post-walk SAM. This sequence was repeated for a second video (eliciting the same emotion). Thus, each emotional state block consisted of 2 emotional induction videos and 2 walking trials. The videos were played inside the virtual environment and the participants were seated during viewing. Participants were asked to describe what emotions they were feeling after each video to validate the emotion elicitation protocol. For walking, participants were instructed to walk using their own self-selected pace and reflect upon the video as they were walking. The SAM was used to collect self-reported data on participant’s current emotional state. This was further used to validate whether the protocol was successful in eliciting the proper emotions alongside their self-reported emotional state. Given the heterogeneity of emotional states induced with the videos, the emotional response on the SAM in combination with the self-reported emotion state was used to confirm correct emotional elicitation in order to include the trial for gait analysis. Small breaks of 5 mins were offered between each block (but not between the video watching to walking portion) to reduce carryover effects of a certain emotion.

Each block was pseudo-randomized for each participant, with the positive emotions of happiness and excitement always being shown before the negative emotions of sadness, anger, and fear. This was due to feedback from a pilot study where participants reported difficulty experiencing positive emotions after having felt negative emotions. Notably, this effect was not reported in the opposite direction (participants did not report issues feeling negative emotions following feeling positive emotions). Five emotional state blocks in total were completed and a post-collection simulator sickness questionnaire was completed to account for any issues with motion sickness caused by the VR environment.

### Gait parameters

Spatiotemporal parameters of gait were measured using the Zeno^TM^ Walkway (ProtoKinetics, LLC, Havertown, USA) gait carpet. The PKMAS software (ProtoKinetics, LLC, Havertown, USA) was used to process and export gait data. During data export, the first and last step made by the participant was excluded to control for the effects of acceleration and deceleration. The 5-factor model of gait, created by Lord et al. [20] was used for gait analysis as it contains clinically relevant parameters which have been largely unexplored in studies investigating emotion and gait (specifically gait variability). All measures of gait variability were calculated using the coefficient of variation (% CV). Table 2 shows the list of all examined gait parameters.

**Table 2:** Gait domains and parameters.

Gait Domain	Gait Parameters
**Pace**	Mean Step Velocity (cm/s)
	Mean Step Length (cm)
	Step Time Variability (%CV)
	Swing Time Variability (%CV)
**Rhythm**	Mean Step Time (s)
	Mean Swing Time (s)
	Mean Stance Time (s)
**Variability**	Step Velocity Variability (%CV)
	Step Length Variability (%CV)
	Step Width Variability (%CV)
**Postural Control**	Mean Step Width (cm)

Shows the list of specific proposed gait parameters intended to be examined. All measures of variability were determined using % coefficient of variation, calculated by the ratio of standard deviation divided by the sample mean.

### Statistical analysis

A one-way repeated measures ANOVA was conducted with the six emotional states representing the within-condition factor for each of the gait parameters listed in Table 2. The assumptions of normality were violated (measured via Shapiro-Wilks test) in at least one condition in all of the gait parameters with the exception of mean step width. Thus, a non-parametric Friedman’s ANOVA was used when assessing differences in gait parameters within all conditions except for mean step width where a parametric one-way ANOVA was used. Durbin-Conover pairwise comparisons or the Student’s t-test were also conducted with Bonferroni’s corrections upon reaching significant result (p<0.05) with the Friedman’s ANOVA or the parametric equivalent, respectively. A Kendall’s (w) value was calculated for effect size estimates and the values between 0.1–0.3, 0.3–0.5, and >0.5 were interpreted as small, medium, and large effect sizes respectively. All statistical test were performed in R Studio with R version 4.2.2. The ggstatsplot package was also used to perform the pairwise comparisons and generate the box plots [32].

Whilst all 36 participants watched almost every video and completed all walking trials, not all participants reported feeling the emotion that was intended. In addition, some participants (13 in total) reported that they were uncomfortable with certain videos and opted out of participating in certain emotional blocks (mainly fear). This resulted in a mismatch between emotional conditions and walking trials assessed across participants, making pairwise and repeated measures analysis a challenge. To account for these individual differences in emotional responses, a sample greater than the formally calculated sample size was recruited and a subset of 23 out of the 36 participants (13 participants removed) that explicitly reported they felt *each* of the targeted emotions was used for analysis.

## Results

Table 1 shows that participants generally did not report feeling extremely positive or extremely negative emotions prior to collection as shown by the moderate positive and negative mDES emotion scores. No statistical difference was also found in the pre and post VRSQ scores.

### Emotion elicitation

The self-reported scores of pleasure and arousal after watching each video were analyzed to validate whether the VR environment and immersive videos did indeed elicit expected emotional responses. Table 3 shows the mean and SD values of the self-reported pleasure and arousal scores and Table 4 shows the results of the pairwise comparisons of the pleasure and arousal scores across all emotional conditions. Generally, positive emotions of happy, excitement had higher mean pleasure scores than the negative emotions of anger, fear, and sadness. In terms of pleasure scores, the only pairwise comparisons that showed no statistical difference were the pairs of neutral-excitement, excitement-happy, anger-fear and fear-sad (p>0.05). From the view of pleasure ratings, the results confirmed that positive emotional conditions did show high ratings in pleasure as was expected.

**Table 3:** Summary results of pleasure and arousal scores across emotional conditions.

Emotion	n	Pleasure (mean ± SD)	Arousal (mean ± SD)
Anger	23	3.04 ± 1.87	5.3 ± 2.29
Excitement	23	7.48 ± 1.95	7 ± 1.83
Fear	23	2.52 ± 1.78	6.7 ± 2.14
Happy	23	8 ± 1.68	5.57 ± 1.93
Neutral	23	6.35 ± 1.3	3.39 ± 2.13
Sad	23	1.61 ± 1.37	4.96 ± 2.74

Shows the mean ± SD of self-reported pleasure and arousal scores across the emotion.

**Table 4:** Wilcoxon test results of each pairwise emotional condition across pleasure and arousal ratings.

		Pleasure	Arousal
group1	group2	p.value	p.value
Neutral	Excitement	0.140	**p<0.001**
Neutral	Happy	**0.011**	**p<0.01**
Neutral	Anger	**p<0.01**	0.109
Neutral	Fear	**p<0.01**	**p<0.01**
Neutral	Sad	**p<0.001**	0.297
Excitement	Happy	0.908	**0.026**
Excitement	Anger	**p<0.01**	**0.042**
Excitement	Fear	**p<0.01**	1.000
Excitement	Sad	**p<0.01**	0.080
Happy	Anger	**p<0.01**	1.000
Happy	Fear	**p<0.01**	0.191
Happy	Sad	**p<0.001**	1.000
Anger	Fear	1.000	**0.035**
Anger	Sad	**p<0.01**	1.000
Fear	Sad	0.102	**0.027**

Shows the results of the pairwise comparisons of pleasure and arousal scores across all emotional conditions and their respective p-values. Pairwise comparison was done using a Wilcoxon rank test with Bonferroni corrections, values bolded to indicate significance.

All emotional conditions except anger and sadness showed an increased arousal score compared to the neutral condition (p<0.05) (Table 4). The excitement condition showed the highest arousal scores, and was statistically greater than the happy (p = 0.026) and anger (p = 0.042) conditions but not the fear and sadness conditions (p > 0.05).

Finally, statistical differences in arousal scores were only observed in the pairwise comparisons of anger-fear (p = 0.035) and fear-sad (0.027). Although arousal scores between emotional conditions were not as distinct as seen in the pleasure scores, generally emotion induction trials did show an increase in arousal scores compared to the neutral condition. Taking into account results from both pleasure and arousal scores, we see expected scores of high pleasure scores for pleasurable emotional conditions and high arousal scores for emotions that were intended to be more arousing (excitement, fear) which were confirmed with these results.

### Gait analysis

Significant differences in gait were only found in the domains of pace and rhythm while gait outcomes from the domains of variability and postural control showed no significant results. Table 5 shows the summary of the mean (SD) of each gait parameter examined across each emotional condition alongside the resultant p value of the Friedman’s tests (with the exception of step width where a one-way parametric ANOVA was used).

**Table 5:** Summary results of gait parameters across emotional conditions.

Gait Parameters	Neutral	Excitement	Happy	Anger	Fear	Sad	Main Effect of Emotional condition (p.value)
Step Length	64.58(6.40)	66.09(6.22)	64.40(5.56)	65.48(5.78)	63.92(6.23)	61.68(6.64)	**p<0.001**
Velocity	122.11(17.99)	128.13(16.99)	121.95(15.59)	126.72(15.09)	123.33(15.88)	113.91(17.35)	**p<0.001**
Step Time	0.53(0.04)	0.52(0.03)	0.53(0.03)	0.52(0.02)	0.52(0.02)	0.55(0.04)	**p<0.001**
Stance Time	0.66(0.06)	0.64(0.05)	0.66(0.05)	0.64(0.04)	0.64(0.04)	0.68(0.06)	**p<0.001**
Swing Time	0.41(0.02)	0.41(0.02)	0.41(0.03)	0.40(0.02)	0.40(0.02)	0.41(0.02)	**p<0.001**
Step Width	9.62(3.44)	9.34(3.03)	9.49(3.27)	9.56(3.40)	9.59(3.36)	9.39(3.59)	0.67
Step Length (%CV)	3.46(0.86)	3.41(0.97)	3.33(1.14)	3.40(0.82)	3.75(1.60)	3.60(1.32)	0.09
Step Time (%CV)	3.17(0.85)	2.93(0.89)	3.01(0.97)	2.77(0.58)	3.20(1.68)	2.96(0.99)	0.52
Velocity (%CV)	4.14(1.38)	3.69(1.18)	3.89(1.32)	3.64(1.10)	3.90(1.74)	3.88(1.47)	0.32
Step Width (%CV)	21.12(9.38)	24.40(11.29)	21.63(8.80)	22.54(10.67)	20.98(8.56)	22.36(9.50)	0.32
Swing Time (%CV)	3.44(1.10)	3.26(1.17)	3.47(1.06)	3.29(0.71)	3.67(1.88)	3.39(1.26)	0.56

Shows the mean (SD) of each gait parameter across the emotional conditions alongside the respective p-value of the one way repeated measures ANOVA. P-values are bolded to indicate significance.

### Pace domain

**Fig 1:**
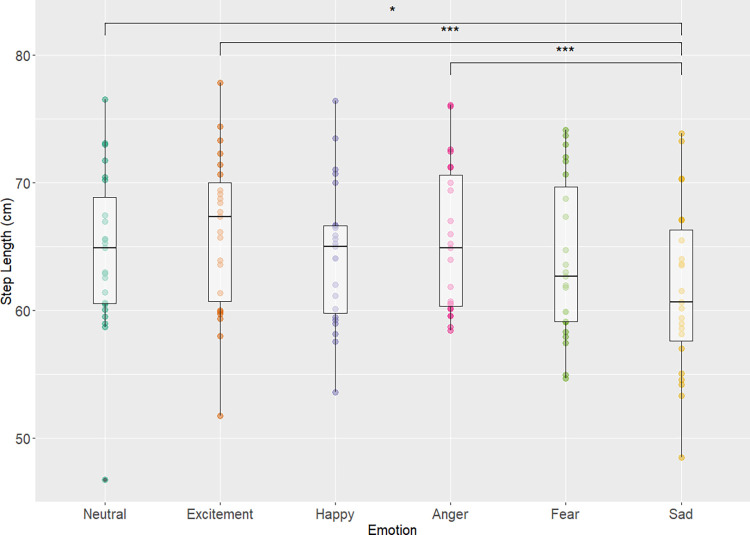
Step length and emotion. Displays the boxplot of each emotional condition and mean step length (cm) for each emotion alongside significant pairwise results. Main effect of emotional condition was found (χ^2^_Friedman_ (df = 5) = 28.39, p = 3.05e^-5^, W_Kendall_ = 0.25) with a small effect size. Adjusted p values are simplified as *, **, *** for p values <0.05, 0.01, 0.001 respectively.

A main effect of emotional condition was found for step length (χ^2^_Friedman_ (*df = 5*) = 28.39, p<0.001, W_Kendall_ = 0.25) with a small effect size (Fig 1). Post hoc analyses showed that step length was significantly reduced during the sadness condition compared to the emotions of anger (p<0.001), excitement (p<0.001), and the neutral emotion (p = 0.02). No other significant differences were observed between the other emotional conditions.

**Fig 2:**
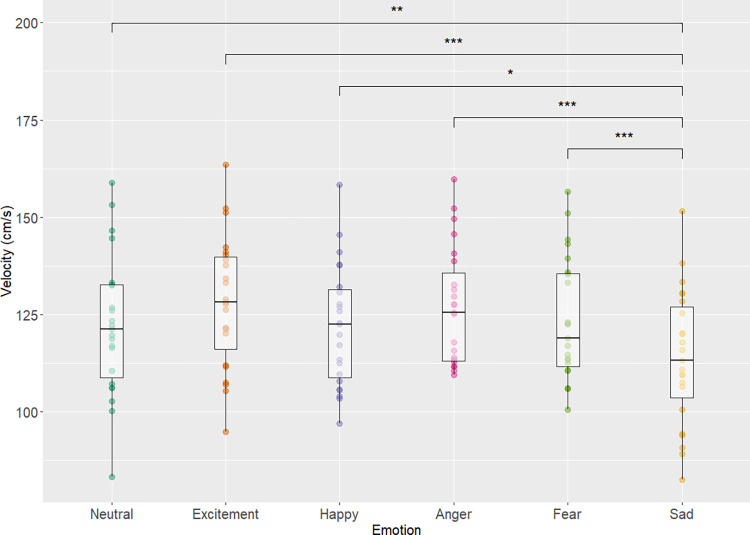
Gait velocity and emotion. Displays the boxplot of each emotional condition and velocity (cm/s) for each emotion alongside significant pairwise results. Main effect of emotional condition was found (χ^2^_Friedman_ (df = 5) = 35.47, p = 1.21e^-6^, W_Kendall_ = 0.31) with a moderate effect size. Adjusted p values are simplified as *, **, *** for p values <0.05, 0.01, 0.001 respectively.

A main effect of emotional condition was also found for gait velocity, (χ^2^_Friedman_ (df = 5) = 35.47, p<0.001, W_Kendall_ = 0.31) with a moderate effect size (Fig 2). Post hoc analysis showed that gait velocity was significantly reduced during the sadness condition compared to the emotional conditions of excitement (p<0.001), anger (p<0.001), fear (p<0.001), neutral (p = 0.00493), and happiness (p = 0.0128). No significant differences in gait velocity were observed when comparing the remaining emotional conditions.

### Rhythm domain

**Fig 3:**
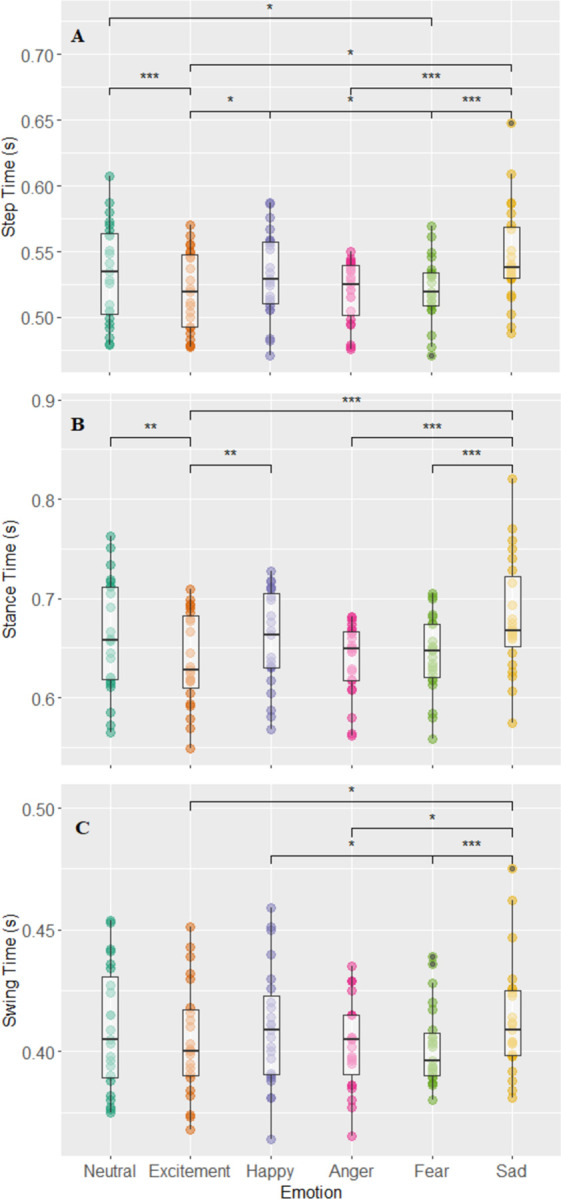
Step time, stance time, swing time, and emotion. Displays the boxplots of each emotional condition and the parameters of step time (A), stance time (B), and swing time (C). Main effects of emotional condition were found in all three parameters of step time χ^2^_Friedman_ (df = 5) = 37.75, p = 4.23e-7, W_Kendall_ = 0.33, stance time χ^2^_Friedman_ (df = 5) = 38.74, p = 2.88e-7, W_Kendall_ = 0.34, and swing time χ^2^_Friedman_ (df = 5) = 23.77, p = 2.41e-4, W_Kendall_ = 0.21. Adjusted p values are simplified as *, **, *** for p values <0.05, 0.01, 0.001 respectively.

Within the rhythm domain, all three gait outcomes (i.e., step time, stance time, and swing time) showed significant results. Fig 3(A) shows a main effect of emotional condition was found for step time (χ^2^_Friedman_ (df = 5) = 37.75, p<0.001, W_Kendall_ = 0.33) with a moderate effect size (Fig 3). Post hoc analysis revealed that participants had greater step time during the sadness condition compared to conditions of fear (p<0.001), anger (p<0.001), and excitement (p = 0.04). Participants walked with decreased step time during the excitement condition when compared to the neutral (p<0.001) and happiness (p = 0.01) conditions. Finally, participants also walked with decreased step time during the fear condition compared to the conditions of happiness (p = 0.04) and excitement (p = 0.04).

A main effect of emotional condition was found for stance times (s) (χ^2^_Friedman_ (df = 5) = 38.74, p<0.001, W_Kendall_ = 0.34), with a moderate effect size (Fig 3B). Post hoc analysis revealed the sadness condition had higher stance times than fear (p<0.001), anger (p<0.001), and excitement (p<0.001) conditions. The excitement condition also had lower stance time than that of neutral (p = 0.0083) and happy (p = 0.02) conditions.

A main effect of emotional condition was found for swing time (s) (χ^2^_Friedman_ (df = 5) = 23.77, p<0.001, W_Kendall_ = 0.21) with a small effect size (Fig 3C). Post hoc analysis revealed the sadness condition displayed longer swing times compared to fear (p<0.001), anger (p = 0.01), and excitement (p = 0.01) conditions. No emotional condition showed any differences to the neutral condition when comparing swing time. The fear condition also showed smaller swing time values compared to the happy condition (p = 0.01).

## Discussion

The primary objective of this study was to examine the influence of the emotional states of happiness, excitement, anger, fear, and sadness on spatiotemporal aspects of gait in a healthy adult non-actor population. The results showed that emotional elicitation method using VR was generally successful in eliciting the expected emotional outcomes as pleasant (happy, excitement) emotions showed higher self-reported pleasure scores compared to unpleasant emotions (sad, fear, anger). Self-reported arousal scores also showed that the emotional videos elicited a greater level of activation in the participants in the excitement, happy, and fear conditions compared to the neutral condition. It was hypothesized that emotions of happy, excitement, and anger would result in increases in gait speed when compared to the *neutral* condition while the opposite was expected for emotions of sadness and fear. This hypothesis was partly supported by the results, the sadness condition did show a decrease in gait speed compared to the neutral, confirming the hypothesis. However, the hypothesis was not supported in the other emotional states, since gait speed was not different from the neutral condition when emotional states such as anger, fear, excitement or happiness were induced. It was also hypothesized that the condition of fear would result in an increase in gait variability compared to the neutral condition, however this was also not supported by the current results as changes to gait variability were not observed across any emotional states. Overall, the results show evidence of emotional states influencing steady state gait in both the pace and rhythm domains. Emotional states may show changes in one domain but not the other as is the case for fear and excitement which showed changes only in the rhythm domain. Sadness showed changes in both domains and was particularly impactful in influencing gait behaviour.

In the pace domain, sadness, above all other emotional conditions showed a large discrepancy in gait parameters such as reductions in step length and gait velocity which is in concordance with previous literature. Barliya et al. showed that, the sadness condition resulted in the slowest gait speed and that fear, happy, and anger conditions showed faster gait compared to sadness, which is reflected in the results of this study [18]. Results also show the condition of sadness had the shortest average step length compared to the other conditions, and effect which is generally supported by previous literature [17] as their results also show sadness having decreased stride length compared to angry, happy and neutral conditions. Current results show that decrease in parameters of gait speed in sadness were similar to decreases in gait speed when comparing healthy controls and patients with major clinical depression. Lemke et al. [6] showed that patients with major depression had a reduction of ~0.23m/s in gait velocity compared to healthy controls. This study showed that sadness condition resulted in difference of ~0.08m/s when compared to the neutral condition. This difference is greater when comparing excitement’s velocity of 1.28 m/s to sadness’ 1.13 m/s which results in a difference of ~0.15 m/s which is closer to the gait speed reduction seen in healthy controls vs patients with clinical depression. Related work that investigated sadness and depression has also shown that there are gait patterns associated with both sadness and depression such as reduced walking speed, arm swing, and vertical head movements and that there are specific gait patterns that are characterized by individuals with a dysphoric mood [5]. While it is not accurate to attempt to equate sadness with major depression, research has shown that sadness is an integral part of depression [33] and thus may be associated with similar gait behaviour. Therefore, the emotion of sadness (as it pertains to gait speed and step length) perhaps could be considered a potential confounder for future gait analysis and research in healthy and clinical populations.

In the rhythm domain, the sadness condition was also different from other emotional conditions (higher stance time and step time compared to excitement, anger, fear) however none of the variables showed a difference between the neutral condition and sadness. These results also are supported by previous literature, as Barliya et al. [18] showed that the sadness condition occupied a lower % swing phase duration and thus the inverse is true where a larger % stance phase duration is observed. Differences in gait between the neutral condition and the conditions of excitement and fear were observed in step time (both excitement and fear conditions showed decreased step times compared to neutral) and stance time (excitement condition lower than neutral). Thus, with these results it is important to highlight that the impact of emotional states on gait may not just be a general modulator of pace and overall gait speed. Compared to the neutral condition, the conditions of fear and excitement, did not show changes in the pace domain but did show changes in the rhythm domain (Figs 1–3), implying that some emotions may only show effect in one domain but not necessarily another From the work of Lord et al. [20] these domains were created to be independent of one another which again may explain why one particular domain is affected by certain emotional conditions but others remain unperturbed. It could be possible that emotional states, especially those of fear and excitement may impact stepping characteristics and the rhythm of gait, independent of pace/speed. Future work could possibly investigate this discrepancy between the two domains and emotions further to understand how one domain may be affected but not the other.

Based on findings from the current study, emotional states did not influence gait variability, nor postural control. Previous research showed that different mood disorders, namely increased anxiety in older adults with PD showed greater step-to-step variability [24] and depression was shown to impact swing time variability (although this exists in the pace domain according to the 5-factor model of gait) [21]. The sample in this study involved young healthy adults. Studies that have investigated dual tasking have shown that an increased cognitive load and cognitive demand consistently have a destabilizing effect on gait on older adults and thus impacting gait variability [34], but the same results are not seen in young adults [35] who demonstrated no change in gait variability in dual task conditions. In concordance with findings from Yogev-Seligmann et al. [35], it is possible that young healthy adults maintain a high degree of automaticity over their gait regardless of emotional conditions leading to fewer changes in their step-to-step variability. It is also possible that perhaps the intensity of emotions elicited in this study were not sufficient enough of a cognitive demand to cause changes in gait variability in young healthy adults.

### Limitations, and future directions

In terms of emotion elicitation, while VR and video was a valid way to elicit emotions, the videos remain still somewhat subjective and are prone to resulting in different interpretations causing inter-participant discrepancy in the SAM reporting and their own self-reported emotions. The usage of the 23-person emotional dataset was an attempt to address this discrepancy in emotional response and reduce the inter-participant variability in report of SAMs and emotional reports. Future studies should consider including a physiological measure of some of these dimensions such as electrodermal skin conductance (EDA) to measure arousal levels, which could give a better indication of the effectiveness of emotion elicitation.

It remains unclear whether the results of this study regarding emotional states and unchanging gait variability parameters would be the same for older adults or a clinical population. Older adults, and patients with depression may walk with increased rumination, resulting in increased cognitive load, and increased distractors as they walk—all which culminates in a modified gait pattern [36,37]. This could potentially introduce more avenues for affective, particularly negative affective states like sadness to further alter gait behavior in this population. The increased cognitive load and fluctuating emotional states (especially negative emotional states) may have a larger impact on gait—similar to that of anxiety and how it affects gait in those with PD. Emotional states such as anxiety can further compromise gait behaviour in older adults with PD given their loss of automaticity and increased requirements for higher order control [24]. We recommend future research be directed towards studying how emotional states affects older adults and those in clinical populations and its similarities to anxiety and other mood disorders.

## Conclusion

The results from this current study show that the emotions of sadness and excitement affect gait in young healthy adults. The main findings show that sadness resulted in smaller steps, reduced gait speed, increased step time and stance times whilst the opposite was observed during excitement. Emotions mainly impacted gait parameters within the pace and rhythm domain, but less so for aspects of gait variability and postural control. It is recommended that future research in both clinical and experimental settings should possibly consider evaluating the emotional state of an individual, particularly the emotions of sadness and excitement as it has shown to influence gait in young healthy adults.

## Supporting information

S1 Checklist STROBE statement—checklist of items that should be included in reports of observational studies. (DOCX)
