# Supplementary material for: Emotional states affect steady state walking performance
Source: PLoS One. 2023 Sep 14;18(9):e0284308. doi: 10.1371/journal.pone.0284308 (PMC10501668; doi:10.1371/journal.pone.0284308)
Supplement: S1 Checklist — (DOCX) [file pone.0284308.s001.docx]

STROBE Statement—checklist of items that should be included in reports of observational studies

|  | Item No. | Recommendation | Page  No. | Relevant text from manuscript |
| --- | --- | --- | --- | --- |
| **Title and abstract** | 1 | (*a*) Indicate the study’s design with a commonly used term in the title or the abstract | 2 | One-way repeated measures ANOVA and pairwise comparisons were used to examine differences in gait parameters across the emotional conditions |
|  |  | (*b*) Provide in the abstract an informative and balanced summary of what was done and what was found | 2 | This study aimed to investigate the influence of emotional states on walking performance to understand whether an emotional state may be an important factor to consider when evaluating gait. Overall, these findings show that in young healthy adults, emotions may impact variety of gait parameters involving pace and rhythm, however have little influence on gait variability and postural control |
| Introduction | | | |  |
| Background/rationale | 2 | Explain the scientific background and rationale for the investigation being reported | 4 | Whilst evidence suggests that mood disorders may give rise to discriminative changes in gait, less work has examined whether similar changes to gait can be induced by alterations in emotional state. Studies have shown that particular emotional states such as happiness and anger feature faster walking with an increased stride length, and increased step count. Much of this past work has focused on quantifying aspects of pace, rather than variability of gait even though recent research has emphasized the clinical relevance of gait variability [11,12]. Furthermore, it is also important to note that most of the previous studies used a sample of professional actors to quantify emotional differences in walking, which can bias or exaggerate effect size of the results given that skilled actors are highly trained to produce stereotyped expressions [8–10]. Thus, there remains a gap in understanding the influence of emotional states on gait behaviour. Given that gait assessments can serve as an important marker for health status, it is imperative to understand the changes in gait characteristics that arise from fluctuations in emotional states; as these changes can possibly confound the gait characteristics observed in both healthy and non-healthy populations, leading to potential false characterizations of gait characteristics. |
| Objectives | 3 | State specific objectives, including any prespecified hypotheses | 5 | The main objective of this study is to measure the effect of emotional states on gait characteristics in healthy young adults with no acting experience using a broader set of gait parameters. It was hypothesized that emotions such as happiness, excitement and anger would result in increased gait speed and step length compared to the neutral control emotion [8–10]. Consequently, emotions such as fear and sadness were expected to result in the opposite, showing decreased gait speed [1,8]. Finally, based on past work in clinical populations, it was also hypothesized that fear would lead to an increase in gait variability. |
| Methods | | | |  |
| Study design | 4 | Present key elements of study design early in the paper | 6,7 | Thirty-six (n = 36) healthy young adult participants from the University of Waterloo were recruited for this study. Participants were given time to explore and navigate the virtual environment to familiarize themselves with the novel stimuli prior to collection. After familiarization was completed, participants performed three neutral walking trials. Walking trials involved 6 passes of walking across the 6m gait carpet resulting in approximately 40-60 total steps captured.  Five emotional states of happiness, excitement, sadness, fear, and anger were elicited in a pseudorandomized blocked design. The blocks started with watching a video, then completing a pre-walk self-assessment manikin (SAM – which evaluated arousal, valence and dominance), followed by the walking trial, and culminating in the post-walk SAM. This sequence was repeated for a second video (eliciting the same emotion). Thus, each emotional state block consisted of 2 emotional induction videos and 2 walking trials. |
| Setting | 5 | Describe the setting, locations, and relevant dates, including periods of recruitment, exposure, follow-up, and data collection | 6 | Thirty-six (n = 36) healthy young adult participants from the University of Waterloo were recruited for this study. [This was not a clinical study; thus, periods of recruitment and follow-up do not apply to this scenario]. |
| Participants | 6 | (*a*) *Cohort study*—Give the eligibility criteria, and the sources and methods of selection of participants. Describe methods of follow-up  *Case-control study*—Give the eligibility criteria, and the sources and methods of case ascertainment and control selection. Give the rationale for the choice of cases and controls  *Cross-sectional study*—Give the eligibility criteria, and the sources and methods of selection of participants |  | Exclusion criteria included any previous difficulty experienced with virtual reality (VR) such as nausea, light-headedness, fatigue etc. Recent history (6 months prior) of physical injuries that impacted gait, use of assistive devices for walking, or clinical diagnosis of mood disorders were also part of the exclusion criteria. Any participants taking medication that may induce or attenuate emotions were also excluded from the study |
|  |  | (*b*) *Cohort study*—For matched studies, give matching criteria and number of exposed and unexposed  *Case-control study*—For matched studies, give matching criteria and the number of controls per case |  | N/A |
| Variables | 7 | Clearly define all outcomes, exposures, predictors, potential confounders, and effect modifiers. Give diagnostic criteria, if applicable | 9 | Table 2 shows the list of all examined gait parameters. |
| Data sources/ measurement | 8* | For each variable of interest, give sources of data and details of methods of assessment (measurement). Describe comparability of assessment methods if there is more than one group | 9 | Spatiotemporal parameters of gait were measured using the ZenoTM Walkway (ProtoKinetics, LLC, Havertown, USA) gait carpet. The PKMAS software (ProtoKinetics, LLC, Havertown, USA) was used to process and export the gait data. During data export, the first and last step made by the participant was excluded to control for the effects of acceleration and deceleration. The 5-factor model of gait, created by Lord et al. was used for gait analysis as it contains clinically relevant parameters which have been largely unexplored in studies investigating emotion and gait (specifically gait variability) |
| Bias | 9 | Describe any efforts to address potential sources of bias | 7 | The emotional blocks were always completed after the neutral walking trials. Each block was pseudo-randomized for each participant, with the positive emotions of happiness and excitement always being shown before the negative emotions of sadness, anger, and fear. This was due to feedback from a pilot study where participants reported difficulty experiencing positive emotions after having felt negative emotions. |
| Study size | 10 | Explain how the study size was arrived at | 6 | G*Power3 (Version33.1, Universitat Dusseldorf, Dusseldorf, Germany) was used to determine sample size using the effect size reported by Halovic & Kroos [9]. Effect size from gait speed was used as the key dependent variable as it is a reoccurring parameter measured across many of the studies. Using an alpha error probability of 0.05, power of 0.8, and the reported effect size of 0.64, a sample size of 18 was calculated [9]. |

Continued on next page

| Quantitative variables | 11 | Explain how quantitative variables were handled in the analyses. If applicable, describe which groupings were chosen and why | 10 |  |
| --- | --- | --- | --- | --- |
| Statistical methods | 12 | (*a*) Describe all statistical methods, including those used to control for confounding | 10 | A one-way repeated measures ANOVA was conducted with the six emotional state conditions representing the within-condition factor for each of the gait parameters listed in Table 2. The assumptions of normality were violated (measured via Shapiro-Wilks test) in at least one condition in all of the gait parameters with the exception of mean step width. Thus, a non-parametric Friedman’s ANOVA was used when assessing differences in gait parameters within all conditions except for mean step width where a parametric one-way ANOVA was used. Durbin-Conover pairwise comparisons or the Student’s t-test were also conducted with Bonferroni’s corrections upon reaching significant result (p<0.05) with the Friedman’s ANOVA or the parametric equivalent, respectively. All statistical test were performed in R Studio with R version 4.2.2. The ggstatsplot package was also used to perform the pairwise comparisons and generate the box plots [18]. |
|  |  | (*b*) Describe any methods used to examine subgroups and interactions |  | N/A |
|  |  | (*c*) Explain how missing data were addressed | 10 | Whilst all 36 participants watched almost every video and completed all walking trials, not all participants reported feeling the emotion that was intended. In addition, some participants reported that they were uncomfortable with certain videos and opted out of participating in certain emotional blocks (mainly fear). This resulted in a mismatch between emotional conditions and walking trials assessed across participants, making pairwise and repeated measures analysis a challenge. To account for these individual differences in emotional responses, a sample greater than the formally calculated sample size was recruited and a subset of 23 out of the 36 participants that explicitly reported they felt each of the targeted emotions was used for analysis. |
|  |  | (*d*) *Cohort study*—If applicable, explain how loss to follow-up was addressed  *Case-control study*—If applicable, explain how matching of cases and controls was addressed  *Cross-sectional study*—If applicable, describe analytical methods taking account of sampling strategy |  | N/A |
|  |  | (*e*) Describe any sensitivity analyses |  | N/A |
| Results | | | | |
| Participants | 13* | (a) Report numbers of individuals at each stage of study—eg numbers potentially eligible, examined for eligibility, confirmed eligible, included in the study, completing follow-up, and analysed | 10 | All participants were eligible but only 23/36 participants were used for full analysis. |
|  |  | (b) Give reasons for non-participation at each stage |  | N/A |
|  |  | (c) Consider use of a flow diagram |  |  |
| Descriptive data | 14* | (a) Give characteristics of study participants (eg demographic, clinical, social) and information on exposures and potential confounders | 6 | Refer to Table 1 |
|  |  | (b) Indicate number of participants with missing data for each variable of interest |  | N/A |
|  |  | (c) *Cohort study*—Summarise follow-up time (eg, average and total amount) |  |  |
| Outcome data | 15* | *Cohort study*—Report numbers of outcome events or summary measures over time |  | N/A |
|  |  | *Case-control study—*Report numbers in each exposure category, or summary measures of exposure |  | N/A |
|  |  | *Cross-sectional study—*Report numbers of outcome events or summary measures |  | N/A |
| Main results | 16 | (*a*) Give unadjusted estimates and, if applicable, confounder-adjusted estimates and their precision (eg, 95% confidence interval). Make clear which confounders were adjusted for and why they were included |  | N/A |
|  |  | (*b*) Report category boundaries when continuous variables were categorized |  | N/A |
|  |  | (*c*) If relevant, consider translating estimates of relative risk into absolute risk for a meaningful time period |  | N/A |

| Other analyses | 17 | Report other analyses done—eg analyses of subgroups and interactions, and sensitivity analyses |  | N/A |
| --- | --- | --- | --- | --- |
| Discussion | | | | |
| Key results | 18 | Summarise key results with reference to study objectives | 16 | The primary objective of this study was to examine the influence of the emotional states of happiness, excitement, anger, fear, and sadness on spatiotemporal aspects of gait in a healthy adult non-actor population. It was hypothesized that emotions of happy, excitement, and anger would result in increases in gait speed when compared to the neutral condition while the opposite was expected for emotions of sadness and fear. This hypothesis was partly supported by the results, the sadness condition did show a decrease in gait speed compared to the neutral, confirming the hypothesis. However, the hypothesis was not supported in the other emotional state conditions, since gait speed was not different from the neutral condition when emotional states such as anger, fear, excitement or happiness were induced. It was also hypothesized that the condition of fear would result in an increase in gait variability compared to the neutral condition, however this was also not supported by the current results as changes to gait variability were not observed across any emotional state condition. |
| Limitations | 19 | Discuss limitations of the study, taking into account sources of potential bias or imprecision. Discuss both direction and magnitude of any potential bias | 18 | …the videos remain still somewhat subjective and are prone to resulting in different interpretations causing inter-participant discrepancy in the SAM reporting and their own self-reported emotions. Future studies should consider including a physiological measure of some of these dimensions such as electrodermal skin conductance (EDA) to measure arousal levels, which could give a better indication of the effectiveness of emotion elicitation. |
| Interpretation | 20 | Give a cautious overall interpretation of results considering objectives, limitations, multiplicity of analyses, results from similar studies, and other relevant evidence | 20 | The results from this current study show that the emotions of sadness and excitement affect gait in young healthy adults. The main findings show that sadness resulted in smaller steps, reduced gait speed, increased step time and stance times whilst the opposite was observed during excitement. Emotions main impacted gait parameters within the pace and rhythm domain, but less so for aspects of gait variability and postural control |
| Generalisability | 21 | Discuss the generalisability (external validity) of the study results | 19 | It remains unclear whether the results of this study regarding emotional states and unchanging gait variability parameters would be the same for older adults or a clinical population. Older adults, and patients with depression may walk with increased rumination, resulting in increased cognitive load, and increased distractors as they walk—all which culminates in a modified gait pattern [21,22]. This could potentially introduce more avenues for affective, particularly negative affective states like sadness to further alter gait behavior in the population. Further work is needed to study how emotional states affects older adults and those in clinical populations. |
| Other information | |  | | |
| Funding | 22 | Give the source of funding and the role of the funders for the present study and, if applicable, for the original study on which the present article is based | 20 | This work was supported by an NSERC Discovery Grant (KEM). |

*Give information separately for cases and controls in case-control studies and, if applicable, for exposed and unexposed groups in cohort and cross-sectional studies.

**Note:** An Explanation and Elaboration article discusses each checklist item and gives methodological background and published examples of transparent reporting. The STROBE checklist is best used in conjunction with this article (freely available on the Web sites of PLoS Medicine at http://www.plosmedicine.org/, Annals of Internal Medicine at http://www.annals.org/, and Epidemiology at http://www.epidem.com/). Information on the STROBE Initiative is available at www.strobe-statement.org.
